# Strong, antioxidant, and biodegradable gelatin methacryloyl composite hydrogel for oxidative stress protection in Schwann cells

**DOI:** 10.3389/fbioe.2025.1586380

**Published:** 2025-06-03

**Authors:** Hongyang Han, Dongcao Ji, Shu Yang, Bo Pang, Xi Chen, Jiaqi Zhu, Wenxin Cao, Tao Song

**Affiliations:** ^1^ NHC Key Laboratory of Cell Transplantation, The First Affiliated Hospital of Harbin Medical University, School of Stomatology, Harbin Medical University, Harbin, China; ^2^ Center for Composite Materials and Structures, Harbin Institute of Technology, Harbin, China; ^3^ Zhengzhou Research Institute, Harbin Institute of Technology, Zhengzhou, China

**Keywords:** gelatin methacryloyl, peripheral nerve injury, tannic acid, Schwann cell, antioxidant

## Abstract

Gelatin methacryloyl (GelMA), a biomaterial widely used in tissue engineering, exhibits excellent biocompatibility and cell adhesion properties. However, its poor mechanical strength and functional monotony restrict broader clinical applications of this material. In this study, we introduced sodium acrylate (SA) and tannic acid (TA) into the GelMA system *via* a two-step crosslinking strategy, successfully fabricating a GelMA/SA–TA (GST) composite hydrogel that achieved dual enhancement of mechanical and antioxidant properties. The incorporation of SA and TA significantly improved the mechanical performance of the hydrogel, which exhibited a maximum tensile modulus of 31.83 ± 2.84 kPa. At the same time, TA endowed the hydrogel with exceptional antioxidant ability, resulting in a free radical scavenging rate of 89.93% ± 0.9% *in vitro*. Biological tests revealed that the GST hydrogel effectively alleviated oxidative stress damage in rat Schwann cells (RSC96) by suppressing the generation of reactive oxygen species (ROS) and promoting the secretion of brain-derived neurotrophic factor (BDNF). This work presents the first report of an antioxidant hydrogel capable of protecting Schwann cells without compromising their mechanical integrity, highlighting its transformative potential for peripheral nerve injury repair. The synergistic SA–TA modification strategy provides new insights into the design of multifunctional biomaterials for neural regeneration applications.

## 1 Introduction

Peripheral nerve injury (PNI) is a prevalent and severe clinical condition that often leads to persistent functional impairment and significant decline in the quality of life of patients, owing to the lack of effective therapeutic strategies (Kouyoumdjian et al., 2017). Although autologous nerve grafting remains the gold standard for PNI treatment, its clinical application is hampered by two major issues: limited donor availability and high rates of postoperative complications (Raza et al., 2020). Schwann cells (SCs), the sole glial cells in the peripheral nervous system, play a pivotal role in neural regeneration, by providing a favorable microenvironment for axonal regeneration (Bosch-Queralt et al., 2023) and also secreting various neurotrophic factors to facilitate nerve repair (Wang et al., 2020). Brain-derived neurotrophic factor (BDNF), a critical member of the peptide growth factor family secreted by SCs, is an indispensable component during neural regeneration (Shi et al., 2022). However, PNI induces excessive accumulation of reactive oxygen species (ROS), resulting in oxidative stress that severely damages SC membranes and organelles, thus compromising the cellular functional integrity and inhibiting nerve repair processes (Kobayashi et al., 2012). Recent advances in biomaterials have made their application a promising approach for nerve injury repair, with growing emphasis on addressing these pathological challenges through innovative material-based solutions.

Among various biomaterials, hydrogels have emerged as prominent candidates for disease diagnosis and therapy. As polymeric materials composed of hydrophilic polymers crosslinked *via* physical or chemical interactions to form three-dimensional networks, hydrogels can absorb and retain substantial water amounts. Their exceptional biocompatibility and tunable physicochemical properties have given them a central role in neural repair research (Han et al., 2024; Hui et al., 2023; Xu W. et al., 2023). However, hydrogels designed for PNI treatment often face challenges such as complex synthesis processes and limited applicability, particularly in the case of antioxidant-functionalized hydrogels, which remain understudied. Gelatin methacryloyl (GelMA), a modified derivative of gelatin, retains the natural triple helix structure of gelatin while incorporating methacryloyl groups into its molecular chains. By adjusting the degree of methacryloylation, GelMA can be engineered to exhibit superior biocompatibility, photocrosslinking ability, and controlled nutrient release properties (Yue et al., 2015). Recent advances in PNI therapy have seen increasing applications of GelMA hydrogels. For instance, Usal et al. developed a novel GelMA–poly (2-hydroxyethyl methacrylate) (pHEMA) hydrogel nerve conduit for peripheral nerve repair, which exhibited favorable porosity and biocompatibility to support the adhesion and proliferation of Schwann cells (Dursun Usal et al., 2019). Similarly, Xu et al. engineered a composite hydrogel (GelMA–CNTF/IGF-1) incorporated into electrospun polycaprolactone (PCL) nerve conduits to repair 15-mm sciatic nerve defects in rats (Xu H. et al., 2023). Nevertheless, the covalent networks formed by pure GelMA under photoinitiation are unstable. The absence of additional functional group modifications results in insufficient mechanical strength and functional limitations, hindering the broader application of pure GelMA in neural regeneration. Furthermore, the simultaneous enhancement of mechanical properties and integration of novel functionalities into GelMA-based systems remains a significant challenge. Thus, the development of composite bioactive hydrogels capable of mitigating oxidative stress, preserving the functionality of Schwann cells, and enhancing the mechanical performance presents both significant therapeutic potential and technical challenges for PNI treatment.

Tannic acid (TA), a naturally occurring polyphenolic compound, exhibits antioxidant, antibacterial, anti-inflammatory, and anticancer properties, which make it widely applicable in biomedical products (Huang et al., 2021). The polyphenolic hydroxyl groups of TA enable it to form hydrogen bonds with carboxyl and amino groups in other hydrogels, stabilizing the molecular architecture of the materials and enhancing their mechanical strength (Liu et al., 2018). Oxidative damage arises from excessive generation of free radicals, which activates cellular oxidative stress pathways. TA has been shown to effectively scavenge free radicals, inhibit protein and lipid peroxidation, and thereby alleviate cellular oxidative stress (Jing et al., 2022). Furthermore, TA promotes the secretion of BDNF (Meine et al., 2024; Park et al., 2011). Recent studies highlight the potential of TA in hydrogel-based therapies; for instance, Fu et al. (Liu et al., 2022) developed a functional hydrogel with *N*-acryloylglycine (NAGA)/GelMA/Laponite/TA composition, which exhibited remarkable anti-inflammatory and antioxidant properties, facilitating spinal cord injury repair in rats. Ning et al. (Zhou et al., 2018) engineered a conductive TA/polypyrrole (PPY) hydrogel, where TA crosslinked PPY chains to achieve gelation, resulting in excellent biocompatibility and high conductivity. Dong et al. (Dong et al., 2022) fabricated a multifunctional TA/NAGA/GelMA hydrogel; hydrogen bonding between TA, NAGA, and GelMA enhanced the mechanical strength while endowing the hydrogel with potent antioxidant activity, resulting in significant potential for bone and cartilage defect repair. These findings indicate that TA incorporation into hydrogels not only improves their mechanical robustness through hydrogen bond-mediated crosslinking, but also endow them with antioxidant capabilities to neutralize ROS. However, reports on TA-integrated GelMA hydrogels for PNI treatment remain scarce, with limited exploration of their ROS suppression mechanisms. Therefore, synthesizing TA-modified GelMA antioxidant hydrogels and investigating their effects on Schwann cells hold substantial scientific and therapeutic value. To address these gaps, in this study we explore the integration of sodium acrylate and TA into the GelMA system. This strategy aims to establish a chemically crosslinked network that can enhance the mechanical performance of the hydrogel while endowing it with ROS-scavenging capabilities.

In this study, we introduced sodium acrylate (SA) and TA into a GelMA system *via* a two-step method, to fabricate a GelMA/SA–TA (GST) composite hydrogel with integrated antioxidant properties. The two-step approach enhanced the crosslinking efficiency, resulting in a uniform TA distribution within the hydrogel matrix. This strategy achieved simultaneous and significant improvements in both mechanical performance and antioxidant capacity. The tensile modulus reached a maximum of 31.83 ± 2.84 kPa, while the radical scavenging rate peaked at 89.93% ± 0.9%. The results of *in vitro* experiments showed that the GST hydrogel effectively mitigated H_2_O_2_-induced oxidative stress in Schwann cells, reducing intracellular ROS levels and promoting the secretion of BDNF. These findings highlight the potential of the GST hydrogel to protect Schwann cells and enhance their functional recovery. The present study provides a critical theoretical basis and technical support for advancing the development of novel biomaterials for nerve repair applications.

## 2 Materials and methods

### 2.1 Materials

GelMA (DS50) was procured from Wenzhou Shuhe Biotechnology Co., Ltd. (China). TA (analytical grade) and SA (analytical grade) were obtained from Tianjin Chemical Reagent Co. (China) and Macklin Biochemical Co., Ltd. (China), respectively. Lithium phenyl(2,4,6-trimethylbenzoyl) phosphinate (LAP, 95%) and 2,2-diphenyl-1-picrylhydrazyl (DPPH) were purchased from Sigma-Aldrich (China) and Yeasen Biotechnology Co., Ltd. (China), respectively. Rat Schwann cells (RSC96) were sourced from Boster Biological Technology Co., Ltd. (China). All reagents were used as received, unless otherwise specified.

### 2.2 Fabrication of GST hydrogels

**FIGURE 1:**
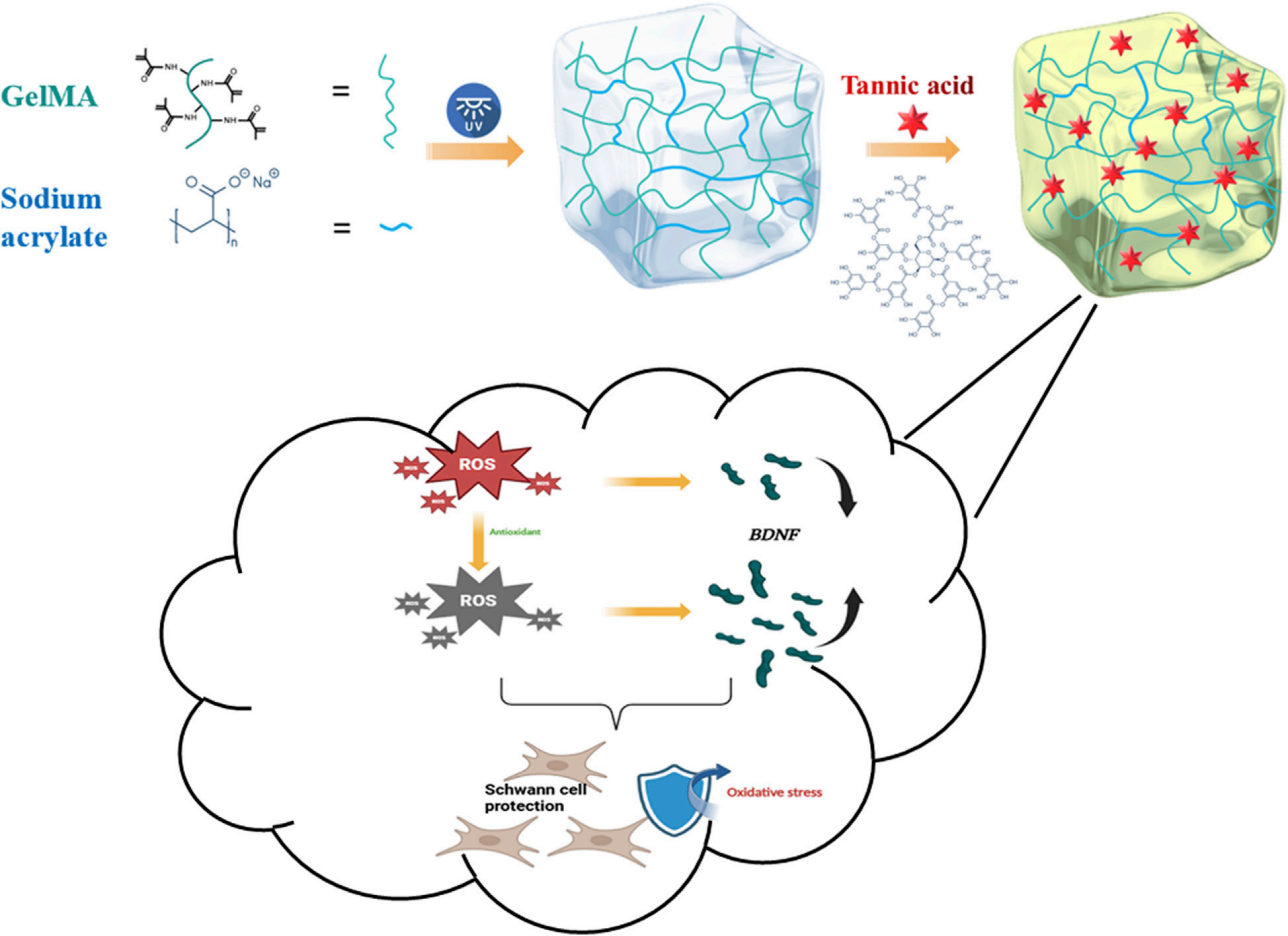
Schematic illustration of GST hydrogel synthesis and its functional effects on Schwann cells.

A two-step crosslinking strategy was employed to synthesize GST hydrogels. First, GelMA and SA were dissolved in deionized water to prepare 10% (w/v) and 2% (w/v) precursor solutions, respectively. The solutions were homogenized by magnetic stirring at 60°C for 30 min. Then, 0.2% (w/v) LAP photoinitiator was incorporated into the mixture under low light conditions. The precursor was then transferred into silicone molds (10 × 10 × 10 mm^3^) and photo-crosslinked under 365 nm UV light (20 mW/cm^2^) for 40 min. Next, the obtained GelMA/SA hydrogels were immersed in TA solutions (5%, 10%, or 20% w/v) for 12 h at 25°C to facilitate TA coordination. After thorough PBS rinsing to remove unbound TA, the final GST hydrogels were designated as GST5, GST10, and GST20, based on the TA concentration (Figure 1).

### 2.3 Physicochemical characterization

#### 2.3.1 Morphological analysis

Lyophilized hydrogels were sputter-coated with gold and imaged using field-emission scanning electron microscopy (FE-SEM; Helios NanoLab 600i, FEI) at a 5 kV acceleration voltage.

#### 2.3.2 FT-IR spectroscopy

Chemical interactions were analyzed *via* Fourier transform infrared (FT-IR) spectroscopy (PerkinElmer Frontier) in the 4,000–500 cm^-1^ range. Samples were prepared by mixing 1% (w/w) test material with KBr pellets.

#### 2.3.3 Water content measurements

Hydrogel samples were weighed (*M*_w_) and then completely dried using vacuum freeze-drying; then, the dried samples were weighed again (*M*_s_). The water content (*W*_c_) was calculated using the following equation:

$$W_{c}\left(\%\right)=\left(Mw-Ms\right)/Msⅹ100\%$$

#### 2.3.4 Swelling tests

Hydrogel samples from different groups were immersed in 8 mL PBS at 37°C for 1, 3, 6, 12, 24, 48, and 72 h. The initial and swollen weights (*W*_0_ and *W*_1_, respectively) were measured at each time point. The swelling degree was calculated as:

$$\mathrm{Swelling degree}\left(\%\right)=\left(W_{1}\;‐\;W_{0}\right)/W_{0}\;ⅹ100\%$$

#### 2.3.5 Degradation tests

To simulate the *in vivo* environment, freeze-dried samples were incubated in 2 μg/mL collagenase type II solution at 37°C for 1, 3, 5, 7, and 10 days. The collagenase solution was replaced every 24 h to maintain the enzyme activity. At each time point, the samples (*n* = 3) were removed, freeze-dried, and weighed (*W*_t_). The remaining mass ratio was calculated as:

$$\mathrm{Remaining mass ratio}\left(\%\right)=W_{t}/W_{0}\;ⅹ100\%$$

#### 2.3.6 Mechanical tests

Uniaxial tensile and compressive tests were performed using a universal testing machine (AGXplus, Shimadzu) operating at 20 mm/min. Elastic moduli were determined from the linear region (10%–20% strain) of stress–strain curves. Cubic samples (10 × 10 × 10 mm^3^) and dog-bone specimens (20 mm gauge length) were used for compression and tensile tests, respectively.

#### 2.3.7 DPPH experiments

The DPPH radical scavenging activity was quantified by incubating the hydrogels (200 mg) in DPPH/ethanol (0.04 mg/mL) for 30 min. The absorbance at 517 nm was measured using a microplate reader (SpectraMax ABS Plus), and the scavenging efficiency was calculated as:

$$\mathrm{Inhibition}\left(\%\right)=\left(1-\frac{A_{x}-A_{b}}{Ac}\right)\times 100\%$$

where A_c_, A_x_, and A_b_ represent the absorbance of the DPPH control solution, the hydrogel-DPPH mixture, and the hydrogel-ethanol background solution, respectively.

#### 2.3.8 TA loading/release profiling

TA exhibits a characteristic UV absorption peak at 276 nm, whereas GelMA/SA displays no absorption at this wavelength. This distinct spectral feature was used to quantitatively determine the TA content by measuring the absorbance at 276 nm. A standard calibration curve for TA was first established within the experimentally relevant concentration range. Cubic hydrogel samples (10 mm × 10 mm × 10 mm) were prepared, weighed (with the weights denoted as *m*_0_), and immersed in 10 mL of TA solutions (5%, 10%, 20%) under sealed, light-protected conditions. The samples were then incubated in a shaking incubator (37°C, 120 rpm) for 12 h. Post-immersion, the TA concentration in the solution was measured using a UV–vis spectrophotometer (Persee, TU-1901), and the absorbed TA mass (*m*_t_) was calculated indirectly. The absorption efficiency was determined using the following equation:

$$\mathrm{Absorbance}\left(\%\right){=m}_{t}/m_{0}ⅹ\mathrm{100}\%$$

For the release efficiency analysis, the total TA content (*m*_1_) within the pre-loaded hydrogel samples was first quantified. The samples were then submerged in 50 mL of PBS, and the release medium was collected at predetermined time intervals. The released TA mass (*m*_r_) was calculated based on the TA concentration in the supernatant, as determined from the UV absorbance at 276 nm and the standard calibration curve. The cumulative release efficiency was calculated as:

$$\mathrm{Release rate}\left(\%\right){=m}_{r}/m_{1}ⅹ\mathrm{100}\%$$

### 2.4 Biological tests

#### 2.4.1 Cytocompatibility assessment

Hydrogel extracts were prepared by immersing sterilized samples (50 mg/mL) in serum-free DMEM for 24 h, followed by addition of 10% FBS. RSC96 cells were cultured in extracts for 1–5 days, with the viability assessed *via* CCK-8 assay (450 nm absorbance). Live/dead staining (calcein-AM/PI) was performed on day 5 and the stained cells were imaged using fluorescence microscopy (Axio Observer 7, Zeiss).

#### 2.4.2 Antioxidant protective effects of hydrogels on schwann cells

CCK-8 assay for cell viability: RSC96 Schwann cells were seeded into 96-well plates at a density of 5 × 10^3^ cells per well and cultured in complete medium for 24 h. Then, the cells were treated for 24 h with one of the following: (1) normal medium (control), (2) H_2_O_2_ (1,000 μM), (3) H_2_O_2_ + GelMA/SA hydrogel extract, or (4) H_2_O_2_ + GST hydrogel extract. The cell viability was assessed using a CCK-8 assay according to the manufacturer’s protocol.

Scratch assay: RSC96 cells were seeded into 24-well plates at 1 × 10^5^ cells per well and allowed to adhere for 24 h. A uniform scratch was created in the cell monolayer using a sterile pipette tip. The cells were divided into four groups: (1) untreated control, (2) H_2_O_2_-treated, (3) H_2_O_2_ + GS, and (4) H_2_O_2_ + GST. The scratch closure was monitored and imaged at 0 and 24 h post-scratch using an inverted microscope (Zeiss Axio Observer 7, Germany). Migration rates were quantified by measuring the residual scratch area with the ImageJ software and using the following equation:

$$\mathrm{Migration efficiency}\left(\%\right)=\left(D_{t}-D_{0}\right)/D_{0}ⅹ100\%$$

where *D*_0_ and *D*_t_ denote the initial and final scratch widths, respectively.

#### 2.4.3 ROS assays

Cells were loaded with 20 μM DCFH-DA after H_2_O_2_/hydrogel treatment. Fluorescence intensities (*Ex*/*Em* = 488/525 nm) were quantified using the ImageJ software.

#### 2.4.4 RT-qPCR analysis

Total RNA was reverse-transcribed using a PrimeScript RT kit (Toyobo), and qPCR amplifications were conducted on a QuantStudio six system using a KOD SYBR Mix kit. The relative BDNF expression was normalized to GAPDH *via* the 2^−ΔΔCT^ method.

#### 2.4.5 Statistical analysis

Data are represented as mean ± SD (*n* ≥ 3). Statistical significance (*p < 0.05, **p < 0.01, ***p < 0.001) was determined by Student’s t-test or one-way ANOVA with Tukey’s post-hoc analysis.

## 3 Results

### 3.1 Structural characterization of GelMA/SA–TA hydrogels

**FIGURE 2:**
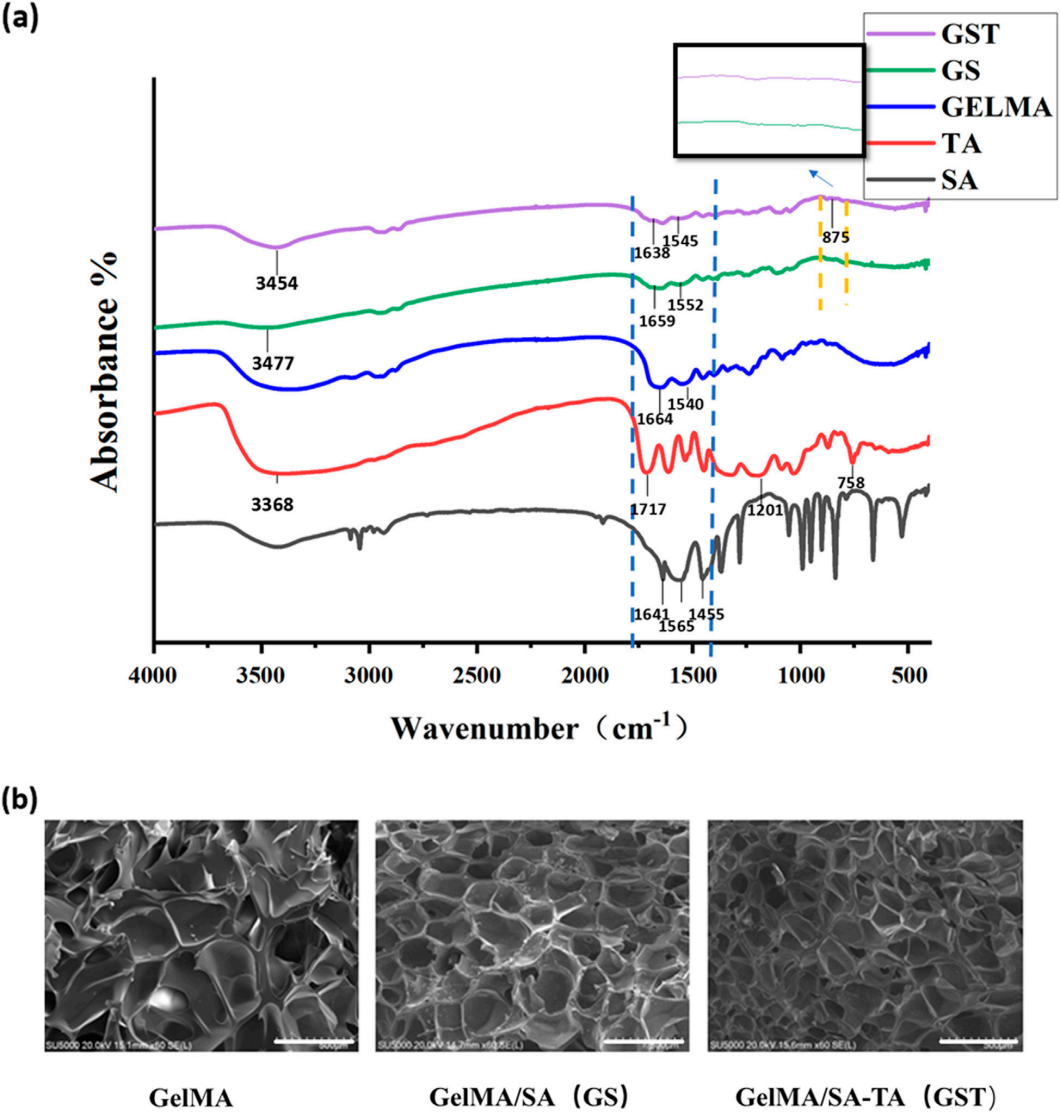
**(a)** FT-IR spectra of individual component materials and composite hydrogels; **(b)** SEM images of hydrogels (scale bar = 500 μm).

The GST hydrogels were prepared according to the experimental procedures described above, and their successful fabrication was confirmed by analyzing the functional groups in the hydrogels using FT-IR spectroscopy (Figure 2a). Characteristic functional groups associated with GelMA, SA, and TA were identified based on previous reports (Gülçin et al., 2010; Huang H. et al., 2024; Magalhaes et al., 2012). For GelMA, the peaks at 1,664 and 1,540 cm^-1^ corresponded to the vibrations of amide C=O and C=C bonds, respectively. In the case of SA, peaks corresponding to the asymmetric and symmetric vibrations of carboxyl C=O groups were observed at 1,565 and 1,455 cm^-1^, while the peak at 1,641 cm^-1^ was attributed to the C=C double bond of the alkene group. For TA, the absorption peak at 1,717 cm^-1^ corresponded to the C=O ester bond, whereas the peaks at 1,201 and 758 cm^-1^ were assigned to the bending vibrations of the benzene ring in TA, and the peak at 3,386 cm^-1^ originated from the hydroxyl group. In the spectra of the GelMA/SA and GelMA/SA–TA hydrogels, the peaks corresponding to the C=O and C=C vibrations of GelMA were present but shifted (blue dashed box), and the intensity of the C=C peak significantly decreased, indicating the formation of a crosslinked network structure. Additionally, the characteristic peaks of TA were shifted: the peak corresponding to the bending vibration of the benzene ring moved from 758 to 875 cm^-1^ (yellow dashed line), whereas the hydroxyl peak shifted from 3,368 to 3,454 cm^-1^, and the C=O peak position of TA changed from 1,717 to 1,638 cm^-1^. The hydroxyl vibration peak shifted from 3,477 cm^-1^ in GelMA/SA to 3,438 cm^-1^ in GelMA/SA–TA, suggesting that TA participated in the hydrogel crosslinking *via* hydrogen bonding. These results confirm the successful preparation of the GelMA/SA–TA composite hydrogel.

The SEM images in Figure 2b reveal that all hydrogel samples exhibited a three-dimensional porous network structure. The addition of SA and TA resulted in an increased pore density and reduced pore size of the composite hydrogels. This phenomenon can be attributed to the covalent bonding between SA and GelMA, as well as the formation of intermolecular hydrogen bonds upon TA incorporation, which collectively enhanced the mechanical properties of the GST hydrogels.

### 3.2 Physicochemical characterization of GelMA/SA-TA hydrogels

#### 3.2.1 Water content and swelling behavior

**FIGURE 3:**
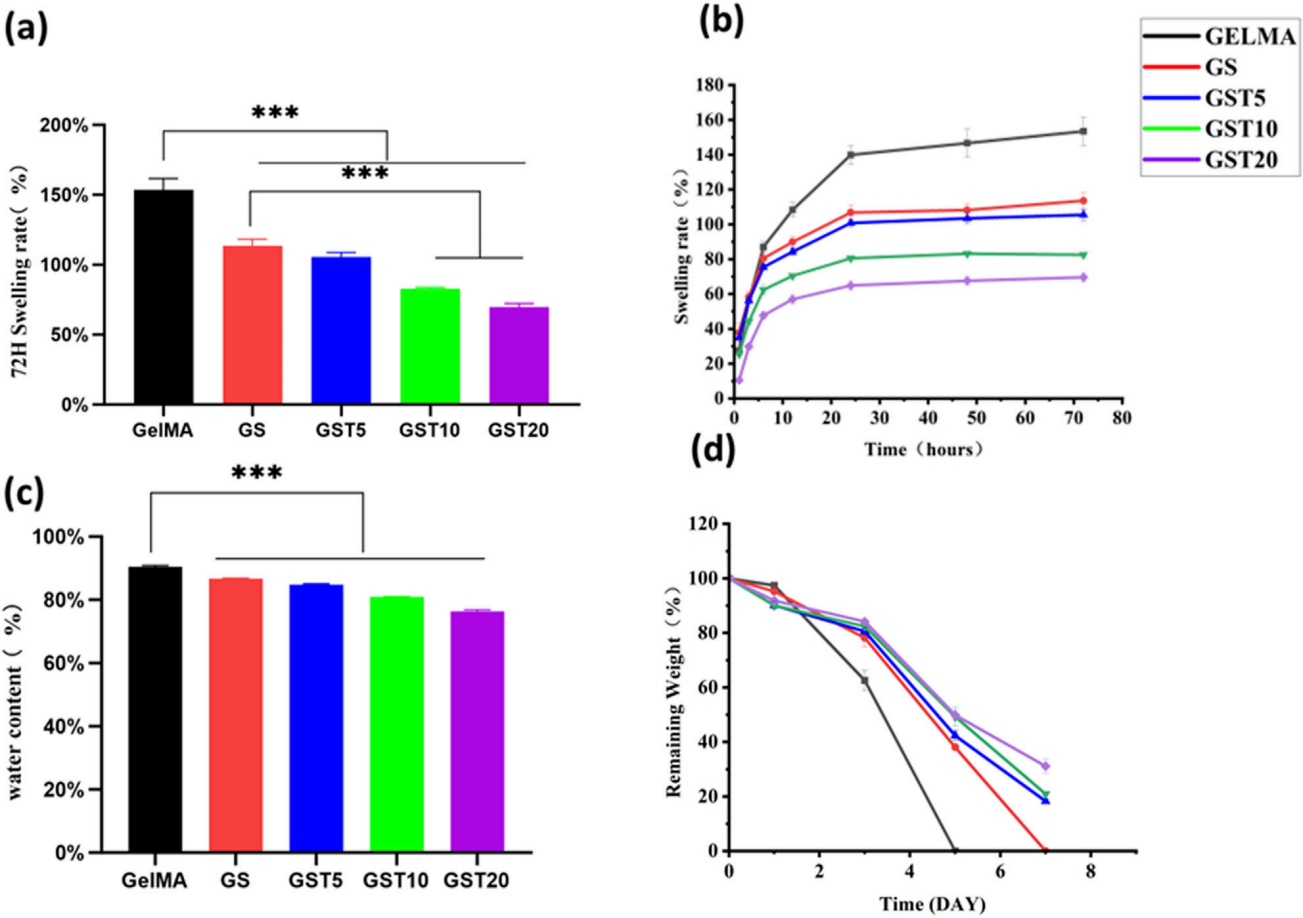
**(a)** Swelling ratios of hydrogels at 72 h; **(b)** time-dependent swelling behavior of hydrogels; **(c)** water content of hydrogels; **(d)** degradation profiles of hydrogels. ***p < 0.001.

A high water content is a critical feature of biological hydrogels, enabling them to retain moisture, flexibility, and elasticity, which are essential properties for *in vivo* applications. The water content of all hydrogels was approximately 80% (Figure 3c). However, compared to pure GelMA, the water contents of GS, GST5, GST10, and GST20 gradually decreased. This effect may be due to the introduction of SA and TA, which reduced the net water content under the same mass, while SA reinforced the hydrogel network and TA increased the number of crosslinking sites. The 80% water content of the hydrogels is close to that of human neural tissue (70%–80%) (Du et al., 2021), facilitating future *in vivo* applications. Additionally, an appropriate swelling capacity is crucial for enabling a biomaterial to adapt to physiological environments. Excessive swelling (>200%) may compress the surrounding tissues. As shown in Figures 3a,b, all hydrogels exhibited moderate swelling, reaching equilibrium at 24 h, with minimal further swelling from 24 to 72 h, owing to the balance between hydrogel network elasticity and osmotic pressure. The equilibrium swelling ratios of the GelMA and GS hydrogels were 153.5% ± 8.1% and 113.6% ± 4.6%, respectively. The incorporation of TA significantly reduced swelling, with GST20 showing the lowest swelling ratio (69.6% ± 2.7%); this indicated that the hydrogen bonds formed by TA led to an increased crosslinking density, thereby hindering water diffusion.

#### 3.2.2 Degradation tests

A controlled degradability is vital to avoid secondary removal in *in vivo* applications. As shown in Figure 3d, all hydrogels exhibited excellent biodegradability in a simulated physiological environment (type II collagenase solution). GelMA was degraded completely within 5 days, while the degradation of GS required 7 days, demonstrating that SA enhanced the network stability. After 7 days, GST5, GST10, and GST20 retained 18.3% ± 0.78%, 20.9% ± 0.55%, and 31.1% ± 2.5% of their initial mass, respectively. The slower degradation of GST20 highlights the role of TA in stabilizing the hydrogel structure through additional crosslinking, ensuring an extended functionality *in vivo* while avoiding long-term retention.

#### 3.2.3 Mechanical properties

**FIGURE 4:**
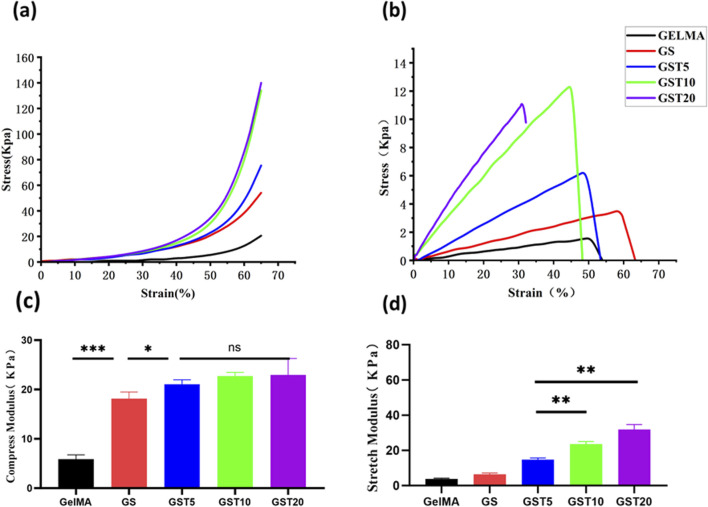
**(a)** Compression tests; **(b)** tensile tests; **(c)** compressive moduli; **(d)** tensile moduli. *P < 0.05, **P < 0.01, ***P < 0.001; ns indicates not statistically significant differences.

The mechanical robustness is a critical property for enabling GelMA-based hydrogels to serve as scaffolds in nerve tissue repair. Compression and tensile tests revealed that SA and TA significantly improved the mechanical performance. Compared to GelMA, GS exhibited a 65% higher elastic modulus and a 18% larger maximum strain. After TA incorporation, the elastic modulus showed a further increase (Figure 4): GST5, GST10, and GST20 exhibited 131%, 273%, and 404% increases relative to GS, respectively. The compressive modulus of GS was 18.13 ± 1.36 kPa, while that of GST20 reached 22.97 ± 3.32 kPa (Figure 4c). In tensile tests, the modulus of GS was found to be 6.33 ± 0.76 kPa, and exhibited a stepwise increase with the TA concentration, peaking at 31.83 ± 2.84 kPa for GST20 (Figure 4d). However, GST20 showed a reduced fracture strength compared to GST10, likely due to uneven crosslinking at high TA concentrations. Overall, GST10 exhibited balanced mechanical properties. Notably, the Young’s modulus of GST10 (31.83 ± 2.84 kPa) was higher than that of a previously reported GelMA/AEPG antioxidant hydrogel (22.2 ± 4.2 kPa) (Mf do et al., 2023) and far exceeded that of pure GelMA (4.1 ± 1.1 kPa).

#### 3.2.4 TA absorption and release

**FIGURE 5:**
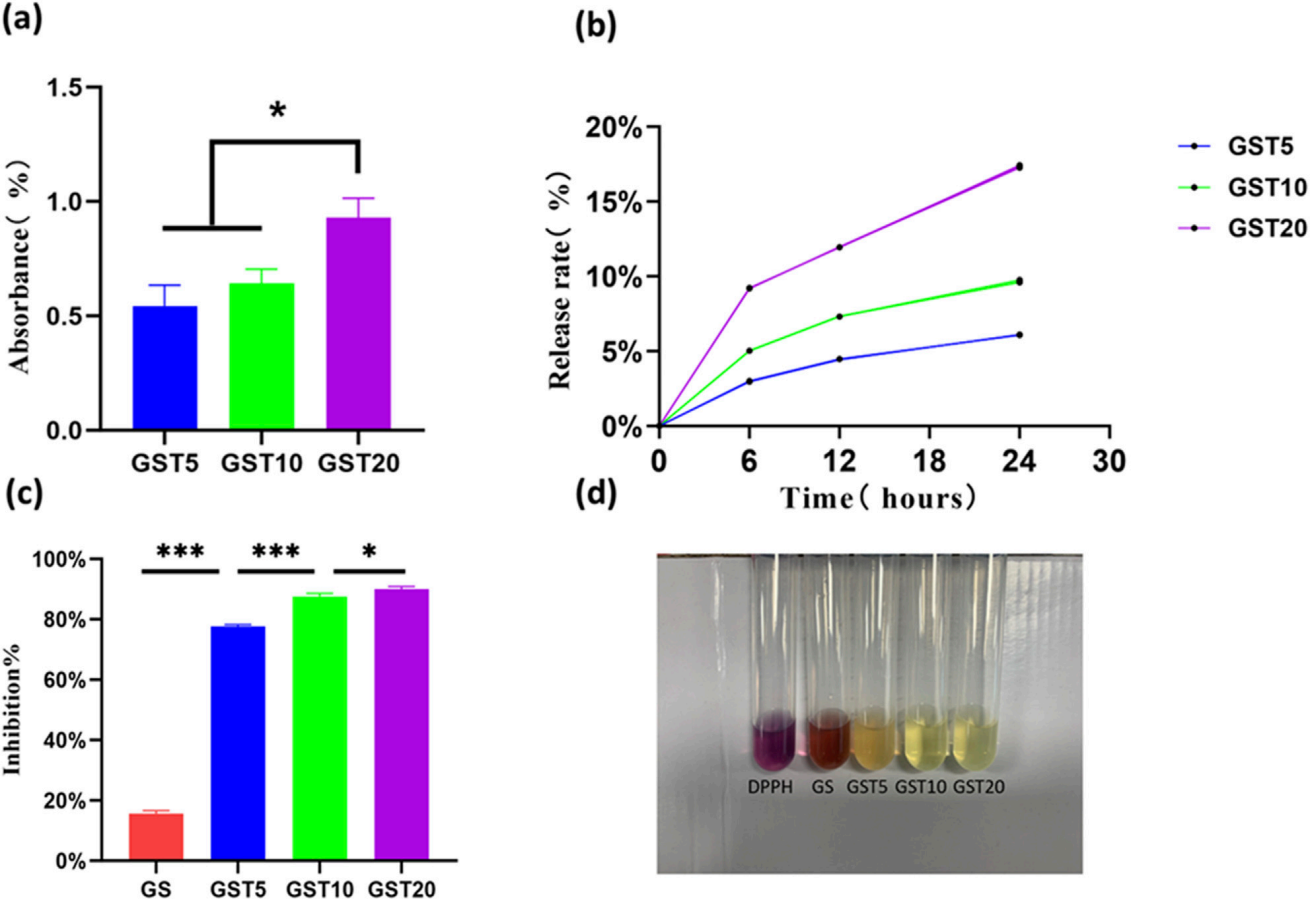
**(a)** TA absorption efficiencies; **(b)** TA release profiles; **(c)** DPPH radical scavenging rates; **(d)** representative images of DPPH assay. *P < 0.05, ***P < 0.001.

TA, which is a natural antioxidant, was incorporated in the hydrogels *via* immersion. Absorption tests showed that GST20 had the highest TA absorption (0.92% ± 0.085%), followed by GST10 (0.6419% ± 0.062%), and GST5 (0.5427% ± 0.091%) (Figure 5a). In release experiments, the TA release rates of GST5, GST10, and GST20 increased over time, reaching 6.089% ± 0.021%, 9.659% ± 0.092%, and 17.35% ± 0.080% at 24 h, respectively (Figure 5b). This sustained release profile highlights the potential of these materials for providing long-term antioxidant effects in nerve injury repair.

#### 3.2.5 DPPH experiments

Antioxidant research represents a pivotal direction in modern scientific and engineering fields, with its core objective being the mitigation of the adverse effects of oxidative reactions on living organisms, materials, and the environment. Hydrogels, widely utilized in applications such as drug delivery and tissue engineering, can achieve a further improved efficacy (for instance, in promoting tissue regeneration) when endowed with antioxidant properties. However, persistent challenges in this context include the insufficient loading and poor stability of antioxidants, as well as complex synthesis protocols. In this study, the introduction of the natural TA compound not only reduced costs, but also ensured both hydrogel stability and efficient antioxidant retention through hydrogen bond-mediated crosslinking. The antioxidant capacity of the hydrogels was evaluated *via* DPPH radical scavenging assays. As shown in Figure 5d, the GST5, GST10, and GST20 hydrogels exhibited significant antioxidant activity, achieving scavenging rates of 77.73% ± 0.5%, 87.57% ± 1.02%, and 89.93% ± 0.9%, respectively (Figure 5c). These values exceed those reported for previously developed antioxidant hydrogels (Gwak et al., 2021; Zeng et al., 2023), underscoring the marked enhancement in antioxidant performance achieved through TA incorporation. Notably, the DPPH scavenging efficiency of the GST hydrogels increased proportionally with the TA content, which can be attributed to the ability of the phenolic hydroxyl groups of TA to neutralize free radicals by donating hydrogen atoms or electrons. The abundance of phenolic hydroxyl groups is directly correlated with the radical scavenging capacity, enabling effective elimination of endogenous ROS generated during PNI and thus reducing oxidative stress damage.

### 3.3 Biological evaluation of hydrogels

#### 3.3.1 Cytocompatibility

**FIGURE 6:**
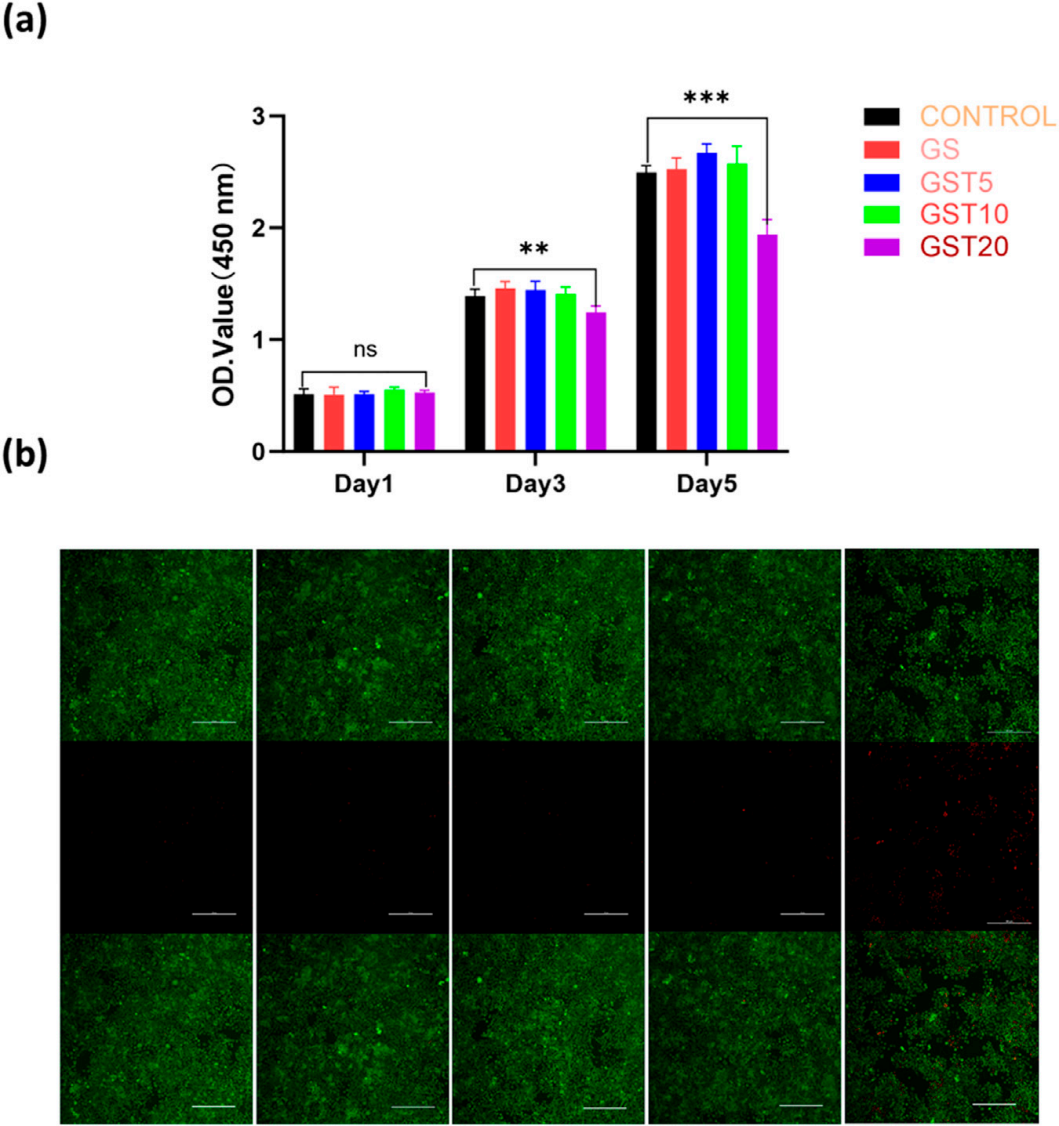
**(a)** OD values from CCK-8 assays of RSC96 cells cultured in different hydrogel extracts for 1, 3, and 5 days; **(b)** live/dead staining of RSC96 cells after 5 days of culture. Green and red colors indicate live and dead cells, respectively (scale bar = 100 μm). **P < 0.01, ***P < 0.001; ns denotes not statistically significant differences.

The evaluation of the biocompatibility of hydrogels is a critical step to ensure their safe application in the biomedical field, with the primary objective being the assessment of their safety toward biological tissues and cells. In this study, the cytocompatibility of the composite hydrogels with RSC96 Schwann cells was comprehensively evaluated using CCK-8 and live/dead staining assays. The RSC96 cells were co-cultured with hydrogel extracts from each group for 1, 3, and 5 days. At day 1, no significant differences in cell viability were observed among the groups. By day 3, however, the GST20 group exhibited reduced cell viability compared to the other experimental groups, and a pronounced decrease was noted at day 5 (Figure 6a), with GST20 cell survival rates dropping below 80%. Live/dead staining (Figure 6b) further revealed minimal numbers of dead cells in the control, GS, GST5, and GST10 groups after 5 days of culture, whereas the GST20 group displayed extensive cell death. These results demonstrate that hydrogels treated with appropriate TA concentrations exhibited no cytotoxicity, whereas an excessively high TA content induced cytotoxic effects that inhibited cell proliferation. Consequently, subsequent cellular experiments employed GST10 as representative group (denoted as “GST” in the following), to balance biocompatibility and functional performance.

#### 3.3.2 Antioxidant protection for schwann cells

**FIGURE 7:**
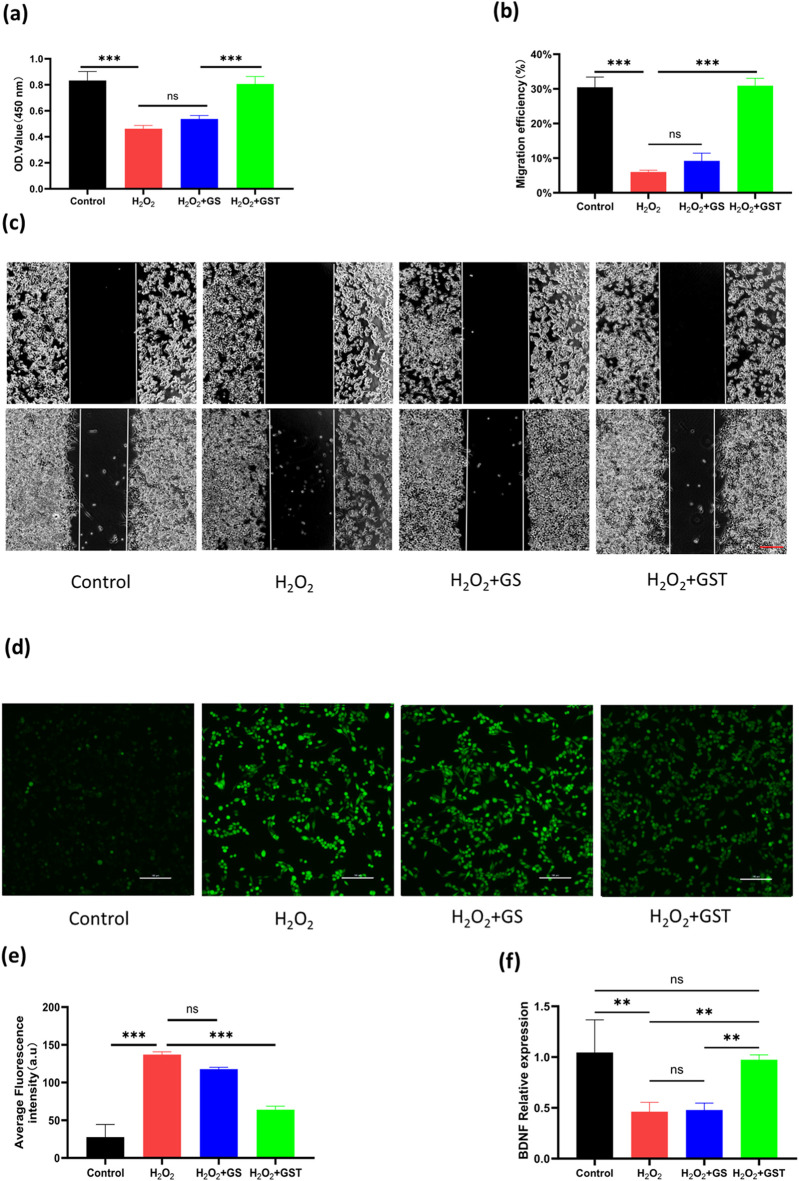
**(a)** OD values of RSC96 cells under H_2_O_2_ induction across different experimental groups; **(b)** quantitative analysis of cell migration rates; **(c)** scratch wound healing assays of control, H_2_O_2_, H_2_O_2_ + GS, and H_2_O_2_ + GST groups (scale bar ; 200 μm); **(d)** ROS staining of cells in each group using DCFH-DA probe; **(e)** quantitative analysis of fluorescence intensity (scale bar ; 100 μm); **(f)** relative BDNF expression levels in control, H_2_O_2_, H_2_O_2_ + GS, and H_2_O_2_ + GST groups. **P < 0.01, ***P < 0.001; ns indicates not statistically significant differences.

ROS play a dual role in cellular processes: at physiological levels, they participate in signaling pathways, whereas excessive accumulation induces oxidative stress and cellular damage. As biomaterials, antioxidant hydrogels can either encapsulate antioxidants or intrinsically modulate intracellular ROS levels to mitigate oxidative injury. Despite the critical role of SCs in peripheral PNI repair, research on antioxidant hydrogels for SC protection remains limited. Thus, developing bioactive hydrogels capable of scavenging ROS in SCs holds significant therapeutic value. In this study, the antioxidant protective effects of hydrogels on SCs were evaluated through CCK-8 assays and scratch wound healing experiments. As shown in (Figure 7a), both the H_2_O_2_ and H_2_O_2_+GS groups exhibited marked decreases in OD values, indicating a suppressed cell proliferation. In contrast, the H_2_O_2_+GST group showed significantly higher OD values compared to the H_2_O_2_ group, suggesting that the application of the GST hydrogel reduced H_2_O_2_-induced cellular damage. These results confirm that TA-containing composite hydrogels effectively inhibited oxidative stress and restore the functionality of SCs under injury-mimicking conditions. In scratch assays (Figures 7b,c), the H_2_O_2_ and H_2_O_2_+GS groups showed reduced cell migration rates relative to the control, whereas the H_2_O_2_+GST group exhibited enhanced migration capacity, further supporting the role of the hydrogel in mitigating oxidative impairment of SC motility. A DCFH-DA fluorescence probe was employed to directly assess the intracellular ROS scavenging efficacy (Figures 7d,e). The strong fluorescence intensity observed for the H_2_O_2_ and H_2_O_2_+GS groups confirmed the successful oxidative stress induction and limited antioxidant activity of the GS hydrogels. Conversely, the H_2_O_2_+GST group displayed a markedly quenched fluorescence, demonstrating its superior ROS neutralization compared to the other groups. Taken together, these findings highlight the strong antioxidant performance of the GST hydrogels in reducing ROS generation and mitigating oxidative stress-induced SC dysfunction, highlighting their potential as advanced therapeutic platforms for nerve repair.

#### 3.3.3 qRT-PCR analysis

BDNF, a critical member of the peptide growth factor family secreted by Schwann cells, plays an indispensable role in neural regeneration. However, oxidative damage suppresses BDNF expression, highlighting the therapeutic importance of simultaneously enhancing BDNF production while mitigating oxidative stress for effective PNI repair. As shown in (Figure 7f), the H_2_O_2_ treatment significantly downregulated BDNF expression in Schwann cells, whereas the application of the GST hydrogel restored the BDNF levels to baseline values. This dual functionality, combining antioxidant activity with BDNF upregulation, makes the application of GST hydrogels a promising strategy to address the multiple challenges of PNI regeneration.

## 4 Discussion

PNI is a clinically prevalent and complex disorder, particularly common in oral and maxillofacial surgery, with facial nerve injury being a representative case. PNI is often characterized by delayed or incomplete self-healing, and conventional therapeutic strategies primarily rely on microsurgical interventions and pharmacotherapy (Lopes et al., 2022). Recent advances in nerve repair biomaterials have introduced promising alternatives to traditional autologous nerve grafting (Hou et al., 2023). Notably, oxidative stress triggered by PNI is recognized as a critical factor hindering functional nerve recovery (Qiu et al., 2019). To address this challenge, we successfully developed a GST composite hydrogel with strong antioxidant properties and conducted systematic cellular tests *in vitro* to assess its therapeutic potential.

The antioxidant properties of the GST composite hydrogel primarily stem from the incorporation of TA, a natural antioxidant capable of efficiently scavenging reactive free radicals (Jing et al., 2022). As demonstrated by DPPH assays, the GST hydrogel exhibited exceptional radical scavenging capacity, exceeding 85%. Similar findings have been reported in previous studies: Park et al. (Gwak et al., 2021) observed that TA-modified hyaluronic acid (HA/TA) hydrogels displayed antioxidant activity, with scavenging rates increasing from 62.2% to 95.3% as the TA concentration rose from 0.5 to 4 mM. Similarly, Guo et al. (Zeng et al., 2023) demonstrated that TA/sodium alginate–phenylboronic acid (SA–PBA) hydrogels outperformed SA–PBA alone in the neutralization of free radicals, with GST hydrogels achieving comparable or superior scavenging efficiency. The antioxidant efficacy of the GST hydrogels was further confirmed by conducting a series of *in vitro* cellular experiments. The results revealed that H_2_O_2_-induced oxidative stress significantly suppressed the proliferation and migration of RSC96 Schwann cells, whereas treatment with the GST hydrogel effectively mitigated these inhibitory effects and restored cellular functionality. DCFH-DA fluorescence staining tests confirmed that the GST hydrogels led to substantially reduced intracellular ROS levels compared to the TA-free GS hydrogels, as evidenced by a weaker green fluorescence intensity (Figure 7d). Although the ROS levels in GST-treated cells did not fully return to the values of the untreated control group, the observed reduction is consistent with the ability of the catechol/pyrogallol groups of TA to neutralize ROS, in agreement with the findings of Ergul et al. (Yulak and Ergul, 2024). qRT-PCR analysis further elucidated the underlying mechanism of the GST-mediated functional recovery. Although the BDNF expression in H_2_O_2_-stimulated Schwann cells cultured with GST hydrogels did not exceed the baseline control levels, it was significantly higher than that in GS hydrogel and H_2_O_2_-only groups, indicating the capacity of TA to mitigate oxidative suppression of BDNF. This result corroborates previous studies highlighting the role of TA in promoting neurotrophic factor expression (Park et al., 2011; Choi et al., 2010). Notably, BDNF is known to upregulate antioxidant enzymes and enhance the expression of neuroprotective antioxidant proteins (Gardiner et al., 2009), suggesting a synergistic mechanism. In summary, the present GST hydrogel exhibits a dual function: it alleviates oxidative stress by scavenging reactive oxygen species, while also enhancing the neural microenvironment by activating neurotrophic factor expression. These properties make GST hydrogels highly promising candidates as next-generation nerve repair biomaterials.

Biocompatibility assessments were performed through cellular experiments. The results demonstrated that GS hydrogels exhibited favorable biocompatibility, while GST hydrogels with varying TA concentrations displayed different cytocompatibility profiles. Specifically, the GST5 and GST10 hydrogels maintained excellent biocompatibility, whereas GST20 (the formulation with the highest TA concentration) exerted a concentration-dependent inhibitory effect on cellular growth (Figure 6). These observations suggest that neither GelMA/SA nor TA inherently induced significant cytotoxicity. However, excessive TA concentrations may suppress cell proliferation, thereby impairing neural repair processes. Although TA possesses antioxidant properties, its overuse can induce toxicity, as evidenced by previous animal studies (Baldwin and Booth, 2022; Doce et al., 2009). Consequently, stringent control of the TA content is imperative during GST hydrogel fabrication and in its future applications for *in vitro* peripheral nerve repair. This optimization ensures that neurotoxic effects caused by excessive TA concentrations are avoided, while preserving the functional efficacy of GST hydrogels.

The physicochemical properties of hydrogels are critical factors for their successful biomedical application. First, a hydrogel must possess a stable architecture to maintain mechanical integrity. As shown in Figure 2b, all hydrogel groups exhibited porous network structures, with an increased structural density observed upon incorporation of SA and TA. Mechanical tests (Figure 4) revealed a significant enhancement in mechanical performance with increasing TA concentrations. Although the compressive moduli exhibited minimal variations among the GST hydrogel groups, all formulations outperformed pure GelMA and GS hydrogels. Notably, the GST10 and GST20 hydrogels possessed markedly higher tensile moduli than the other groups. However, GST20 exhibited reduced fracture strength compared to GST10, likely due to the inhomogeneous crosslinking induced by excessive TA concentrations, creating localized mechanical weak points. Future studies should focus on optimizing the TA concentration in order to balance mechanical robustness and structural homogeneity. To ensure their efficacy *in vivo*, hydrogels must also exhibit controlled degradability. Under simulated physiological conditions (type II collagenase solution), all hydrogels gradually degraded over 3–7 days (Figure 3d). The GST hydrogels displayed slower degradation rates compared to the GelMA and GS groups, which could be attributed to the TA-enhanced crosslinking density and network stability. This modification preserved the biodegradability while prolonging the antioxidant efficacy *in vivo*. The porous structure of the GST hydrogels, as observed *via* SEM, promoted a high water retention (Figure 3c) and an efficient fluid absorption/release. This structural feature also enabled controlled TA absorption and sustained release, consistent with Niu et al.‘s findings (Liu et al., 2024) showing that gradual TA release from hydrogels contributed to ROS scavenging and anti-inflammatory effects, properties also found for our GST system. Swelling experiments (Figure 3b) showed a concentration-dependent reduction in swelling ratios with increasing TA content, consistent with Sun et al.‘s report (Huang Y. et al., 2024). This behavior stems from TA-mediated increases in crosslinking density, which restrict water diffusion. The reduced swelling mitigates mechanical compression on adjacent tissues, enhancing the practical applicability of GST hydrogels in the nerve repair field. In parallel with similar GelMA-based composite hydrogels, such as the TA/N-acryloyl glycine (NAGA)/GelMA hydrogel developed by Dong et al. (Dong et al., 2022), which enhances mechanical strength and reduces swelling ratio through hydrogen bonding interactions between TA, NAGA, and GelMA, our system shares structural similarities. Both designs leverage TA-mediated hydrogen bonding to reinforce hydrogel crosslinking, thereby exhibiting enhanced physicochemical properties.

In this study, a novel multifunctional composite hydrogel was fabricated using a two-step synthesis method. Preliminary experiments revealed that direct addition of TA into the hydrogel precursor solution resulted in self-polymerization of its phenolic hydroxyl groups, thereby suppressing the photocrosslinking process under UV irradiation. To address this issue, a two-step immersion protocol was adopted, which effectively prevented self-polymerization while promoting hydrogen bonding interactions between TA and GelMA/SA. This approach resulted in enhanced mechanical performance, reduced swelling ratios, and lower degradation rates, consistent with previous methods (Hsu et al., 2020). Furthermore, TA, as a natural antioxidant, imparted additional functional benefits to the hydrogel. However, the two-step synthesis remains relatively laborious, and future studies should focus on streamlining the fabrication process in order to improve its scalability. Systematic characterizations showed that the GST10 hydrogels exhibited optimal mechanical properties, favorable biocompatibility, and exceptional antioxidant activity, making them promising candidates for PNI repair applications. These findings highlight the potential of the GST10 hydrogels to address critical challenges in neural regeneration, providing a robust platform for further translational development.

## 5 Conclusion

The GelMA/SA–TA composite multifunctional hydrogel developed in this study was successfully applied to investigate its protective antioxidant effects on Schwann cells. The results demonstrated that RSC96 cells co-cultured with GST hydrogels exhibited superior resistance to H_2_O_2_-induced oxidative stress damage compared to those in the GS hydrogel and blank control groups. The incorporation of TA significantly enhanced the mechanical properties of the GST hydrogels, while other physicochemical characteristics, such as degradability and swelling ratio, also showed excellent performance. These findings highlight the immense potential of GST hydrogels for future development and clinical applications. However, to better address the challenges associated with PNI repair, it is essential to obtain a deeper understanding of the mechanisms underlying nerve damage (especially oxidative stress-related pathways) and of the roles of various components involved in neural regeneration. Further *in vivo* animal studies are needed to confirm the efficacy of GST hydrogels and understand their independent and synergistic effects in nerve regeneration. Such investigations will provide critical insights to advance the development of promising therapeutic strategies for clinical treatment of peripheral nerve injuries.

## Data Availability

The raw data supporting the conclusions of this article will be made available by the authors, without undue reservation.
